# Protective Effects of Biscoclaurine Alkaloids on Leukopenia Induced by ^60^Co-*γ* Radiation

**DOI:** 10.1155/2020/2162915

**Published:** 2020-05-18

**Authors:** Min Wang, Xueheng Xie, Yuyang Du, Guoxu Ma, Xudong Xu, Guibo Sun, Xiaobo Sun

**Affiliations:** ^1^Beijing Key Laboratory of Innovative Drug Discovery of Traditional Chinese Medicine (Natural Medicine) and Translational Medicine, Institute of Medicinal Plant Development, Peking Union Medical College and Chinese Academy of Medical Sciences, 100193 Beijing, China; ^2^Research Center on Life Sciences and Environmental Sciences, Harbin University of Commerce, 150076 Harbin, China

## Abstract

**Objective:**

Leukopenia, a common complication of tumor chemoradiotherapy, contributes serious damage to the hematopoietic, gastrointestinal, and immune systems of the body and can cause delay, discontinuation, or even failure to tumor treatment, thereby greatly threatening human health. The present study aims to investigate the protective effects of biscoclaurine alkaloids (BA) on leukopenia.

**Methods:**

This study was conducted on 60 Kunming mice, which were randomly divided into six groups containing 10 animals each. A hematology analyzer was used to count white blood cells (WBC) in the peripheral blood cell. Mice serum was collected, and the granulocyte-macrophage colony-stimulating factor, vascular cell adhesion molecule 1 (VCAM-1), and interferon-*γ* (IFN-*γ*) were detected by enzyme-linked immunosorbent assays. Pathological changes were detected through hematoxylin and eosin staining in the liver and spleen of mice. The spleen and liver ultrastructures were observed via electron microscopy.

**Results:**

Results showed that BA ameliorated WBC, PLT reduction in the peripheral blood and significantly increased the levels of IFN-*γ* and VCAM-1 in mice serum. BA reduced ionizing radiation-induced injuries to spleen, mitigated the reduction of superoxide dismutase (SOD), and significantly decreased the malonaldehyde (MDA) and xanthine oxidase (XOD) levels in the liver.

**Conclusion:**

BA enhanced the immune and hematopoietic functions and ameliorated the oxidative stress induced by ^60^Co-*γ* radiation, revealing its therapeutic potential both as a radioprotector and as a radiation mitigator for leukopenia induced by ^60^Co-*γ* radiation.

## 1. Introduction

Leukopenia is a disease mainly induced by chemoradiotherapy and is characterized by the continuous reduction in peripheral leukocytes to less than 4500 cells/mm^3^ [1]. The reduction in leukocytes results in decreased immune functions and the occurrence of serious infections in various parts of the body, which are mostly on the respiratory, digestive, and urogenital tracts. With these infections, hyperpyrexia, mucosal necrotizing ulcerations, septicemia, and septicopyemia may arouse, placing human health in serious danger. The use of granulocyte colony-stimulating factor (G-CSF), pegylated G-CSF, and granulocyte-macrophage CSF (GM-CSF) has been approved by the United States Food and Drug Administration for treating radiation injuries, such as acute myelogenous leukemia, neutropenia, and leukopenia [2, 3]. However, their applications are associated with high treatment costs and numerous side effects, such as fever, myalgia, bone pain, and erythema [3]. Thus, the identification of cheap, effective, and low-toxicity products against ionizing radiation (IR) induced leukopenia is urgent.

IR results in DNA damage and the formation of reactive oxygen species (ROS), which can lead to serious damage in hematopoietic, immune, and antioxidant functions [4–6]. Hematopoietic stem/progenitor cells (HSC/HPC) are responsible for hematopoietic recovery and extremely sensitive to IR. Exposure to IR decreases the numbers of HSCs and HPCs and causes apoptosis and mitotic arrest [7]. Immune cells are sensitive to IR. The immune-suppressive effect of high doses of IR, which increases apoptosis in all splenocyte subpopulations, is well approved [8]. The liver is rich in antioxidant enzymes and mitochondria, wherein ROS is primarily produced [9]. IR induces excessive ROS in the liver and causes oxidative stress, an imbalance between oxidant and antioxidant agents. It is through this mechanism that IR severely damages the antioxidant capacity of the liver.

Biscoclaurine alkaloids (BA) were extracted from *Stephania cepharantha* Hayata, a well-established Chinese herb traditionally used to treat a number of diseases, such as parotitis, gastric ulcer, and leukopenia [10]. Modern researches have proven their therapeutic potential for several diseases [11–13], such as leukocyte sequestration during cardiopulmonary bypass [14], cancer management [14], alopecia areata [15], and venomous snakebites [16]. The combination of four BA (cepharanthine, berbamine, isotetrandrine, and cycleanine) increases the recruitment of polymorphonuclear cells from the marginal pool [12]. Such combination also reduces not only radiation-induced apoptosis but also the cytotoxic effects of radiation on T-cell proliferation. These findings indicate that BA have extraordinary protective effects on radiation-induced leukopenia. Several studies have reported the protective effects of BA on leukopenia, but their mechanism is still not fully explored. In the present study, we focused on the protective effects of BA on the reduction of leukocytes, oxidative stress, and immune injuries induced by IR.

## 2. Materials and Methods

### 2.1. Radiation

A cobalt radiation source at the Institute of Radiation Medicine, Academy of Military Medical Sciences (Beijing, China), was used for irradiation. Unanesthetized animals were given a 250 cGy total body irradiation (TBI) at a rate of 95.61 cGy/min. Sham irradiated mice underwent the same procedures as *t* irradiated mice.

### 2.2. Reagents

BA (log: 20150624, containing, Supplementary Figure 1, Table 1) was provided by Anhui Jiufang Pharmaceutical Research Institute Co. LTD (Anhui, China). Recombinant human G-CSF injection was obtained from Qilu Pharmaceutical Co. LTD (Shandong, China). Enzyme-linked immunosorbent assay (ELISA) kits for GM-CSF, IFN-*γ*, and VCAM-1 were purchased from RayBiotech (Peachtree Corners, USA). Detection kits for SOD, MDA, and XOD were purchased from Nanjing Jiancheng Bioengineering Institute (Nanjing, China). BA used in this study refers to the compound containing tetrahydropalmatine, palmatine, and roemerine.

### 2.3. Experimental Animals

Male Kunming mice used in this study weighed 18–22 g and were purchased from SPF (Beijing) Laboratory Animal Technology Co., Ltd. and using only male mice was to avoid the data variability that might be caused by the estrous cycle of female mice. All of the mice were housed in a specific pathogen-free facility, maintained at 23°C–25°C with a 12 h day/12 h dark cycle throughout the study, and provided with autoclaved food and sterile water in the Laboratory Animal Center of the Institute of Medicinal Plant Development, Peking Union Medical College and Chinese Academy of Medical Sciences (Beijing, China). All studies were performed in accordance with procedures approved by the Institute of Medicinal Plant Development Animal Care and Use Committee (IACUC).

### 2.4. Animal Grouping and Treatments

**Figure 1:**
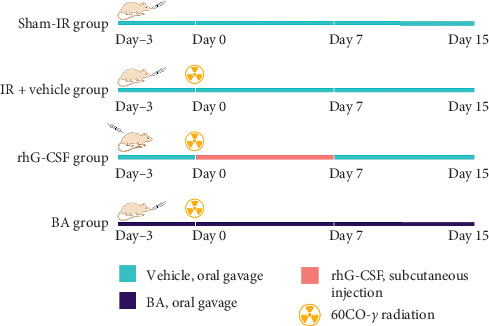
Animal treatments. Notes: Mice in the sham-IR group were given an oral gavage with the same volume of vehicle once a day from day −3 to day 15, and did not expose to ^60^Co-*γ* radiation. IR + vehicle group received the same volume of vehicle by oral gavage once a day from day −3 to day 15, and exposed to ^60^Co-*γ* radiation at day 0. The rhG-CSF group was exposed to the radiation at day 0 and was subcutaneously injected with rhG-CSF 1 h after the radiation, once a day for 7 days; and the group was given an oral gavage with the same volume of vehicle once a day from day −3 to day 0 and from day 7 to day 15. Mice in the BA group received multiple doses of BA by oral gavage, once a day, from day −3 to day 15. Vehicle: the same volume of 1% carboxymethyl cellulose, once a day, by oral gavage. BA: biscoclaurine alkaloids, at 5, 10, 20 mg/kg, once a day, by oral gavage. rhG-CSF: recombinant human G-CSF, at 100 *μ*g/kg, once a day, by subcutaneous injection.

A total of 60 male Kunming mice were housed as previously described for at least 1 week to allow them to adjust to the new environment before use. They were randomly assigned by weight to the following groups (*n* = 10/group): (1) Sham-IR, (2) IR + vehicle, (3) Low dose BA (L-BA), (4) Middle dose BA (M-BA), (5) High dose BA (H-BA), and (6) Recombinant human G-CSF (rhG-CSF). All groups except the sham-IR group were irradiated with 2.5 Gy ^60^Co-*γ* radiation via TBI. Mice in L-BA, M-BA, and H-BA groups were given an oral gavage with BA (5, 10, 20 mg/kg, respectively) once a day for 3 days before the IR and 15 days after the IR. Mice in the sham-IR group were given the same volume of 1% carboxymethyl cellulose as a vehicle by oral gavage once a day for 3 days before and 15 days after the IR. Mice in the rhG-CSF group were given an oral gavage with the same volume of vehicle once a day for 3 days before the IR and were subcutaneously administered with rhG-CSF (100 *μ*g/kg) once a day for 7 days after the IR. Graphical summary of animal treatments is available for easy understanding (Figure 1) .

### 2.5. Peripheral Blood Routine Examination

Blood (40 *µ*L) was directly collected from the tail tip and added with 160 *µ*L blood thinner. The solution was mixed gently on a rotary shaker until analyses were done for white blood cells (WBC), erythrocytes, and platelets using a hematology analyzer (Sysmex XT-2000i, Japan).

### 2.6. Determination of Immune Cell Cytokines and Cell Adhesion Molecules

After blood collection on the 15th day, blood was drawn by removing the eyeballs, and then it was centrifuged at 3000 rpm for 15 min. The serum was collected and the expressions of GM-CSF, IFN-*γ*, and VCAM-1 in the bone marrow matrix were determined using the ELISA kit.

### 2.7. Histopathological Examination of the Spleen and Liver

After the experiment, mice were sacrificed. Spleen and liver tissues were collected, fixed in 4% formaldehyde, embedded in paraffin, cut in 5 mm, and stained with hematoxylin and eosin (HE) for histopathological examination under an optical microscope.

### 2.8. Ultrastructure Examination of the Spleen and Liver

Spleen and liver tissues were collected, cut into 1 mm^3^, and fixed in 2.5% glutaraldehyde. After paraffin embedding and ultrathin section (30–40 nm), the ultrastructure of cells was observed via transmission electron microscopy (TEM).

### 2.9. Liver Antioxidant Capacity Test

After the execution of mice, their livers were collected. Cold 0.9% NaCl solution was added to prepare 10% (w/v) liver tissue homogenate. The homogenate was centrifuged at 4000 rpm for 10 min at 4°C. The supernatant was harvested and stored at −80°C for detecting SOD, XOD, and MDA.

### 2.10. Statistical Analysis

All data were presented as mean ± standard error of mean (SEM). Different groups were compared via 2-tailed unpaired Student's *t*-tests using GraphPad Prism 8.0 software (GraphPad Software Inc., San Diego, CA, USA). Graphical presentation of data was performed with GraphPad Prism. A *P*-value of less than 0.05 was considered statistically significant.

## 3. Results and Discussion

### 3.1. BA Alleviated TBI-Induced WBC Reduction in Peripheral Blood

**Figure 2:**
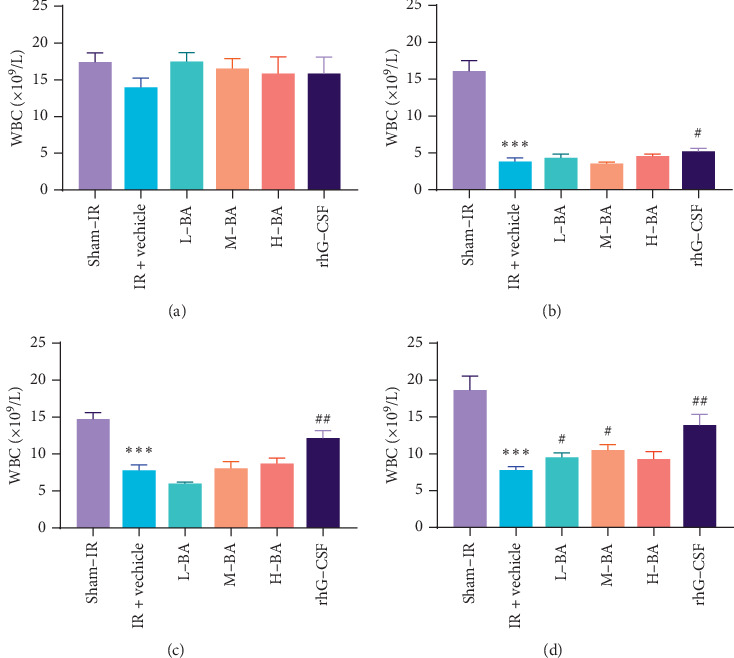
Effects of BA on peripheral WBC (leucocyte) in mice (mean ± SEM, *n*=10). Notes: (a) Peripheral WBC of mice at day −3. (b) Peripheral WBC of mice at day 3. (c) Peripheral WBC of mice at day 7. (d) Peripheral WBC of mice at day 15. ^*∗*^*P* < 0.05, ^*∗∗*^*P* < 0.01, and ^*∗∗∗*^*P* < 0.001 compared with the sham-IR group at the same time point; ^#^*P* < 0.05 and ^##^*P* < 0.01 compared with the IR + vehicle group at the same time point.

Before TBI, the WBC count of mice showed no significant difference (*P* > 0.05) among groups (Figure 2(a)). At days 3 and 7, WBC in all BA groups showed no significant difference (*P* > 0.05) compared with IR + vehicle group, but WBC in rhG-CSF group significantly increased compared with IR + vehicle group (*P* < 0.05) (Figures 2(b) and 2(c)). At day 15, the WBC count in IR + vehicle exhibited a significant decrease compared with that in the sham-IR group (*P* < 0.05) (Figure 2(d)). At day 15, the WBC count in the L-BA, H-BA group (*P* < 0.05), and the rhG-CSF group (*P* < 0.01) increased significantly compared with that in IR + vehicle group (Figure 2(d)).

### 3.2. BA Alleviated TBI-Induced Platelets Reduction in Peripheral Blood

**Figure 3:**
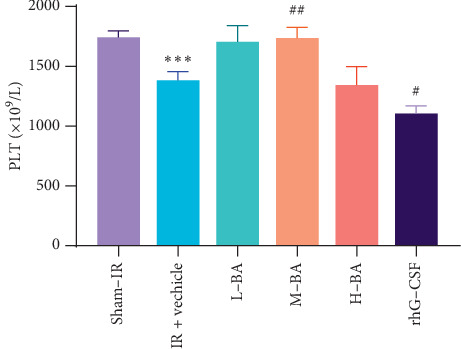
Effects of BA on peripheral Platelets in mice (mean ± SEM, *n* = 10). Notes: Peripheral PLT of mice at day 15. ^*∗*^*P* < 0.05, ^*∗∗*^*P* < 0.01, compared with the sham-IR group; ^#^*P* < 0.05, ^##^*P* < 0.01, compared with the IR + vehicle group.

At day 15, the peripheral PLT of the IR + vehicle group significantly decreased compared to the sham-IR group (*P* < 0.01) (Figure 3). Compared with the IR + vehicle group, M-BA group significantly increased the PLT level (*P* < 0.01) (Figure 3). While the PLT level of the rhG-CSF group significantly decreased (*P* < 0.05) (Figure 3).

### 3.3. BA Increased the Level of Cell Cytokines and Cell Adhesion Molecules

**Figure 4:**
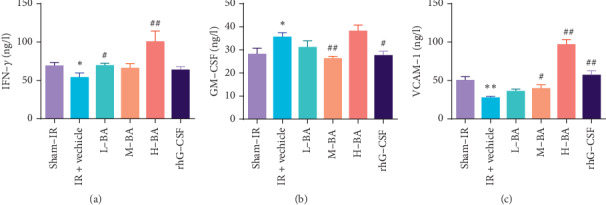
Effects of BA on peripheral cytokines and cell adhesion molecules (mean ± SEM, *n* = 10). Notes: (a) Peripheral IFN-*γ* level. (b) Peripheral GM-CSF level. (c) Peripheral VCAM-1 level. ^*∗*^*P* < 0.05 and ^*∗∗*^*P* < 0.01 compared with the sham-IR group; ^#^*P* < 0.05 and ^##^*P* <  0.01 compared with the IR + vehicle group.

Compared with the sham-IR group, the IR + vehicle group significantly reduced the IFN-*γ* level (*P* < 0.05) (Figure 4(a)). Compared with the IR + vehicle group, L-BA (*P* < 0.05) and H-BA (*P* < 0.01) group significantly increased the IFN-*γ* level in the serum; but the IFN-*γ* level in M-BA and the rhG-CSF group showed no significant differences compared to the IR + vehicle group (*P* > 0.05) (Figure 4(a)). Compared with the sham-IR group, the IR + vehicle group significantly increased the GM-CSF level (*P* < 0.05) (Figure 4(b)). Compared with the IR + vehicle group, GM-CSF level in the L-BA and H-BA group showed no significant changes (*P* < 0.05), but the GM-CSF level in M-BA and rhG-CSF group significantly decreased (*P* > 0.05) (Figure 4(b)). Compared with the sham-IR group, the IR + vehicle group significantly increased the VCAM-1 level (*P* < 0.05) (Figure 4(c)). Compared with the IR + vehicle group, VCAM-1 level in the L-BA group showed no significant changes (*P* < 0.05), but the GM-CSF level in the M-BA, H-BA, and rhG-CSF groups significantly increased (*P* < 0.05) (Figure 4(c)).

### 3.4. Effects of BA on Histopathologic Changes of the Mice Spleen

**Figure 5:**
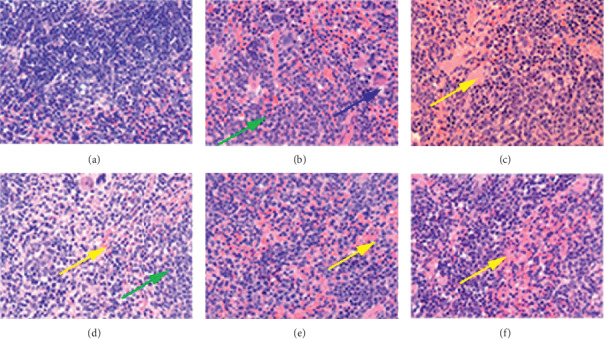
Effects of BA on spleen tissues of ^60^Co-*γ*-irradiated mice stained with HE. (a) Sham + IR. (b) IR + vehicle. (c) rhG-CSF. (d) L-BA. (e) M-BA. (f) H-BA. Sections were stained with HE and viewed at a magnification of ×100. Green arrow: white pulp atrophy. Yellow arrow: red pulp myeloid cells proliferation. Blue arrow: vacuolation.

The boundaries of white and red pulps of the spleen in the sham-IR group were clear and without evident pathological changes, and the red pulp showed mild extramedullary hematopoiesis (Figure 5(a)). In comparison with the sham-IR group, the lesions of the spleen in the IR + vehicle group were mainly manifested as atrophy of the white pulp, decreased lymphocytes in the white pulp, sparse lymphatic nodules, and unclear boundaries between the red and white pulps (Figure 5(b)). Lymphocyte apoptosis, the cavernous structure in the red and white pulps (Figure 5(b)). In comparison with the IR + vehicle group, the M-BA, H-BA, and rhG-CSF groups enhanced the proliferation of red pulp myeloid cells and alleviated white pulp atrophy and lymphocyte apoptosis (Figures 5(c), 5(e), and 5(f)).

### 3.5. Effects of BA on the Spleen Ultrastructure

**Figure 6:**
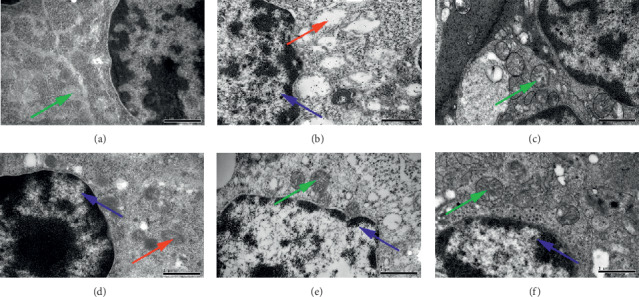
Effects of BA on the spleen ultrastructure. (a) Sham-IR. (b) IR + vehicle. (c) rhG-CSF. (d) L-BA. (e) M-BA. (f) H-BA. Sections were viewed at a magnification of ×30,000. Green arrow: mitochondrial with normal mitochondrial cristae and complete membrane. Red arrow: mitochondrial with swelling, vacuoles, or even degeneration. Blue arrow: chromatin condensation and margination.

The spleen tissue, which is highly sensitive to IR, carries out hematopoietic and immune functions in mice. Therefore, we observed the subcellular structure (or molecular level) changes of the spleen tissue via TEM in order to deepen the understanding of the damage in tissues and cells induced by radiation and the protective mechanism of drugs. In the sham-IR group, the nucleus region was clear; the nucleus was big and round, and abundant heterochromatins were observed in it. Intact cell membrane, a few mitochondria with clear cristae, intact rough endoplasmic reticulum were also observed (Figure 6(a)). In the IR + vehicle group, splenocytes showed morphological changes of apoptosis. Chromatin condensation and margination, nuclear fragmentation, or even dissolution were observed. Besides, rough endoplasmic reticulum was broken, mitochondria swelled or degenerated (Figure 6(b)). In the rhG-CSF group, intact cell membrane, large nucleus, abundant heterochromatins, and clear karyotheca were observed. Some intact mitochondria with continuous cristae were found in the cytoplasm (Figure 5(c)). In the L-BA group, chromatin margination and mitochondria with swelling and vacuoles were observed. The nucleus was big and round; the nucleus region was clear (Figure 6(d)). In the M-BA and H-BA group, some swelling mitochondria were found, but most mitochondria were normal and with clear cristae. Chromatin margination was also observed. (Figures 6(e) and 6(f)). The ultrastructure of splenocytes in the M-BA and H-BA group was improved clearly. The results revealed BA could alleviate the spleen damage induced by radiation.

### 3.6. BA Improved the Antioxidant Capability of the Mice Liver

**Figure 7:**
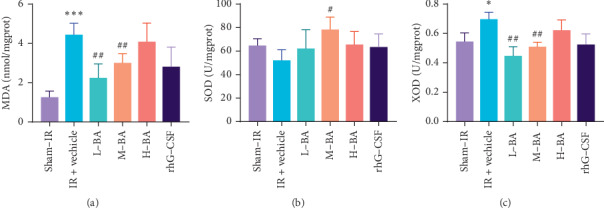
Effects of BA on the antioxidant capacity of ^60^Co-*γ*-irradiated mice (mean ± SEM, *n* = 10). Notes: (a) MDA level in mice liver. (b) SOD level in mice liver. (c) XOD level in mice liver. ^*∗*^*P* < 0.05, ^*∗∗*^*P* < 0.01, and ^*∗∗∗*^*P* < 0.001, compared with the sham-IR group; ^#^*P* < 0.05, ^##^*P* < 0.01, and ^###^*P* < 0.001, compared with the IR + vehicle group.

Compared with the sham-IR group, the IR + vehicle group significantly increased the MDA and XOD levels (*P* < 0.05) (Figures 7(a) and 7(c)). Compared with the IR + vehicle, the L-BA and H-BA groups significantly reduced the MDA and XOD levels (*P* < 0.01) (Figures 7(a) and 7(c)). Meanwhile, compared with the IR + vehicle group, the SOD level significantly increased in the M-BA group (*P* < 0.05) (Figure 7(b)). But, there were no significant differences between the sham-IR group and the IR + vehicle group (*P* < 0.05) (Figure 7(b)). The results suggested that BA could improve the antioxidant capability damage in the liver caused by IR.

### 3.7. Effects of BA on Histopathologic Changes of the Mice Liver

**Figure 8:**
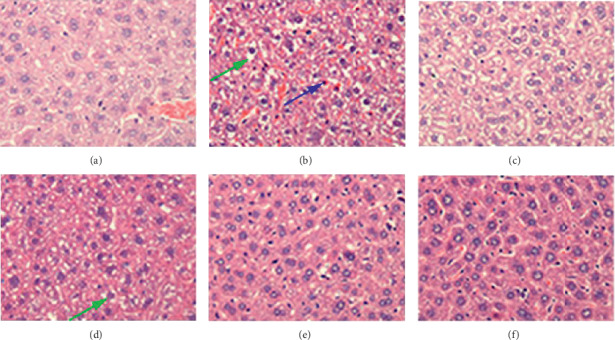
Effects of BA on liver tissues of ^60^Co-*γ*-irradiated mice stained with HE. (a) sham + IR. (b) IR + vehicle. (c) rhG-CSF. (d) L-BA. (e) M-BA. (f) H-BA. Sections were stained with HE and viewed at a magnification of ×100. Green arrow: cellular swelling. Blue arrow: karyopyknosis.

The observation of the pathological changes in livers of different experimental groups showed that the main pathological manifestation of the liver was hepatocyte swelling, which might be associated with external or internal factors (Figure 8). The hepatopathy in the IR + vehicle group, compared with the sham-IR group, was mainly severe degeneration and swelling of hepatocytes, cytoplasmic vacuolation, partly karyopyknosis, and even vanishing (Figure 8(b)). Hepatocellular swelling was mildly ameliorated in the H-BA group compared with the IR + vehicle group (Figure 8(f)). The hepatocellular swelling in the G-CSF group showed insignificant changes compared with that in the IR + vehicle group (Figure 8(c)).

### 3.8. Effects of BA on the Liver Ultrastructure

**Figure 9:**
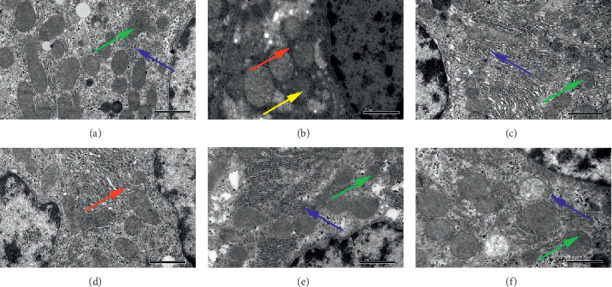
Effects of BA on the liver ultrastructure. (a) Sham-IR. (b) IR + vehicle. (c) rhG-CSF. (d) L-BA. (e) M-BA. (f) H-BA. Sections were viewed at a magnification of ×30,000. Green arrow: mitochondrial with normal mitochondrial cristae and complete membrane. Yellow arrow: mitochondrial with swelling, vacuoles, or even degeneration. Red arrow: rough endoplasmic reticulum fracture. Blue arrow: normal rough endoplasmic reticulum.

In the sham-IR group, mitochondrial with intact membrane and clear cristae, rough endoplasmic reticulum and glycogen granules were observed in the cytoplasm. Intact nuclear bilayer and clear nuclear pore were also found (Figure 9(a)).

In the IR + vehicle group, many hepatocytes were damaged; nuclear deformation, chromatin condensation, and widened perinuclear space were observed. Besides, there were also cytoplasmic vacuolization, rough endoplasmic reticulum fracture in the cytoplasm (Figure 9(b)).

In the rhG-CSF group, the morphology of hepatocytes and cell nuclei were close to the sham-IR group. The mitochondria damage was alleviated, and mitochondria membrane and cristae were clear. Cell nucleus was intact, nuclear membrane and nuclear pore were clear to see, and abundant heterochromatin was observed in the nucleus (Figure 9(c)).

In the L-BA group, cells had an ultrastructure similar to the rhG-CSF group. Slightly deformed nucleus, parts of cristae were unclear and decreased, and fractured rough endoplasm reticulum was observed. Nucleoprotein particles in the rough endoplasm reticulum were severely detached; nucleus and nuclear membrane were intact and clear to see (Figure 9(d)). The M-BA group had similar ultrastructure to the rhG-CSF group. The nucleolus area was visible, heterochromatin was increased, and part of the cristae was visible (Figure 9(e)). The H-BA group had an ultrastructure similar to the L-BA group. Only part of the cristae was visible and the intranuclear heterochromatin decreased (Figure 9(f)). The results indicated that BA could improve the ultrastructure damage caused by IR.

## 4. Discussion

Radiotherapy is an effective strategy for targeting tumor tissues, but it may induce injuries in normal tissues [17, 18]. Hematopoietic tissues are sensitive to IR [19–21]. When the body is exposed to IR, peripheral blood changes occur due to the reduction of the proliferation and division of blood cells. WBC in peripheral blood are derived from bone marrow pluripotent hematopoietic stem cells [3]. Their changes in quantity and quality can reflect the functional state of bone marrow hematopoietic tissues [22–24]. Therefore, the number of WBC in peripheral blood is considered to be an important marker for evaluating radiation injury. The present study showed that the WBC count in the peripheral blood of IR-exposed mice significantly decreased. BA at 5 and 10 mg/kg significantly upregulated the peripheral WBC. The results suggested that BA can alleviate IR-induced WBC reduction. It was reported that Cepharanthin® (CE), a medicine with multiple therapeutic potentials, could accelerate recovery from leukopenia induced by IR in mice; interestingly, CE comprises biscoclaurine alkaloids extracted from *Stephania cepharantha* Hayata, too. BA used in this study refers to the compound containing tetrahydropalmatine, palmatine, and roemerine. However, BA in CE was cepharanthine, berbamine, isotetrandrine, and cycleanine [12]. Though with different chemical compositions, both CE and BA showed potent activity of upregulating peripheral WBC in mice exposed to IR.

Though rhG-CSF could ameliorate the WBC reduction induced by IR with great potency, it decreased the PLT level in a dose-dependent manner [25]. Even worse, a more severe thrombocytopenia might be caused by G-CSF combined with chemotherapeutic drugs to treat cancer patients compared with only chemotherapy treatment [26]. Consistently, it was found that rhG-CSF aggravated the PLT reduction induced by IR at day 15 in this study. However, BA (10 mg/kg) showed compelling efficacy against IR-induced PLT induction, which may greatly reduce the risk of hemorrhage, infection, and even death caused by thrombocytopenia.

The spleen not only plays a critical role in prompting the recovery of mice from chemotherapy- or radiation-induced hematopoietic suppression through hematopoiesis stimulation [9, 27] but also regulates the immune system. Increased cellularity of the white and red pulps in the BA groups indicated that BA perform radioprotection by alleviating spleen injury induced by IR. We further examined the protective capability of BA on immune injury by examining the levels of several important cytokines. IFN-*γ* is a cytokine secreted by activated T lymphocytes (type II interferon), which plays an important regulatory role in the immune response. This cytokine not only activates macrophages but also promotes the differentiation of cytotoxic T cells (CTLs) [28]. VCAM-1 plays a key role in leukocyte recruitment [29], making it a key determinant for immune functions. BA (5, 10 mg/kg) upregulated IFN-*γ* level significantly; BA (10, 20 mg/kg) significantly increased VCAM-1 level. These results indicate that BA enhances immune functions by prompting leukocyte recruitment and leukocyte cytokine production and enhancing immune responses.

ROS is often considered a critical mediator to radiation injury, which subsequently damages biomacromolecules, such as DNA and proteins [30]. Therefore, targeting ROS is regarded as an effective strategy for ameliorating radiation injury. Recent studies have proven that ATP-sensitive potassium channel (K_ATP_ channel), especially the mitochondrial membrane ATP-sensitive potassium channel (mK_ATP_ channel), is associated with the generation of intracellular ROS [31]. Meanwhile, the liver is one of the tissues in which the K_ATP_ channel is highly expressed. Thus, the liver is the main tissue attacked by ROS, and liver injury after radiation must be examined. SOD is a primary antioxidant enzyme in cells [4]. MDA is the final product of lipid peroxidation, and its level reflects the extent of damage [32]. XOD generates oxidants by shuttling electrons derived from purine oxidation to reduce O_2_ either univalently (O_2_^•−^) or divalently (H_2_O_2_). The elevation of XOD activity may result in increased formation of reactive nitrogen species (RNS) via the diffusion-limited reaction between XOD-derived O_2_^•−^ and •NO to generate ONOO^−^ [33]. Hence, SOD, MDA, and XOD are important indices for measuring the antioxidant capacity of organisms. In our study, BA protected mice against TBI-induced damage by suppressing the cellular levels of MDA and XOD and by increasing the expression of SOD. These results suggest that BA can scavenge free radicals and ROS via redox balancing, resulting in reduced oxidative stress in mice. In addition, histopathological and ultrastructural observation results of the liver confirmed that BA have a regulatory effect on maintaining the integrity of liver cell structure in TBI-induced mice.

In our study, BA showed no dose-response in WBC regulation and some other readouts. However, BA at 10 mg/kg showed significant effects on most of the tested indicators. Therefore, BA at 10 mg/kg was recommended for further study and developing as a radioprotector.

## 5. Conclusions

Our data suggested that BA could attenuate the IR-induced leukopenia and thrombopenia by increasing WBC and PLT level, enhancing immune functions, and upregulating antioxidant capacity. It has great potential to be developed as a radioprotector and as a radiation mitigator.

## Data Availability

The data used to support the findings of this study are available from the corresponding author upon request.

## Supplementary Materials

Supplementary Materials Figure 1: the HPLC fingerprint of the biscoclaurine alkaloids and its main components. Table 1: HPLC quantification of biscoclaurine alkaloids. Table 2: effects of BA on histopathologic and morphological changes of the spleen. Table 3: effects of BA on histopathologic and morphological changes of the liver.
