# Supplementary material for: Protective Effects of Biscoclaurine Alkaloids on Leukopenia Induced by 60Co-γ Radiation
Source: Evid Based Complement Alternat Med. 2020 May 18;2020:2162915. doi: 10.1155/2020/2162915 (PMC7251465; doi:10.1155/2020/2162915)
Supplement: Supplementary Materials — Figure 1: the HPLC fingerprint of the biscoclaurine alkaloids and its main components. Table 1: HPLC quantification of biscoclaurine alkaloids. Table 2: effects of BA on histopathologic and morphological changes of the spleen. Table 3: effects of BA on histopathologic and morphological changes of the liver. [file 2162915.f1.pdf]

Supplementary materials

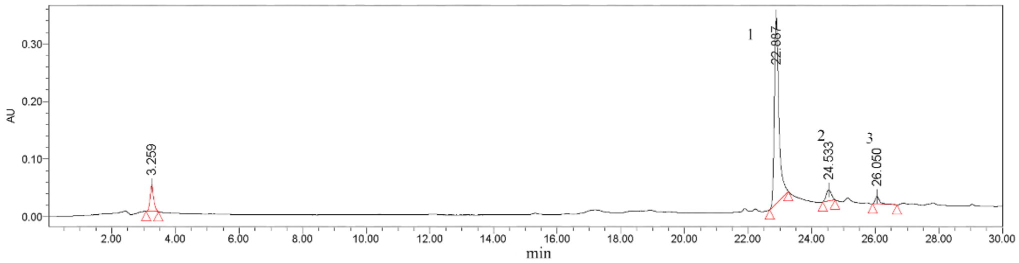

Figure 1: The HPLC fingerprint of the Biscoclaurine Alkaloids and its main components. And name of the compounds: tetrahydropalmatine (1), palmatine (2), roemerine (3).

Table 1: HPLC quantification of Biscoclaurine Alkaloids

Peak	Chemical name	t_R(min)
1	tetrahydropalmatine	5.545
2	palmatine	6.552
3	roemerine	6.982

Table 2: Effects of BA on Histopathologic and Morphological Changes of Spleen

Group	Animal number	Extramedullary hematopoiesis	White pulp atrophy	Lymphocyte apoptosis
**sham-IR(A)**	A1	+ +	-	-
	A2	+ +	-	+ +
	A3	-	-	-
	A4	-	-	-
	A5	-	-	-
	A6	-	-	-
	A7	-	-	-
	A8	-	-	-
**IR+vehicle(B)**	B1	-	+ +	-
	B2	-	-	-
	B3	-	+	+ +
	B4	-	+ +	-
	B5	-	+ +	+ +
	B6	+ +	+ +	+
	B7	+ +	-	+
	B8	+ + + +	+	+
**L-BA(C)**	C1	+ +	+	-
	C2	-	+	-
	C3	-	-	-
	C4	+ +	++	-
	C5	-	-	-
	C6	+ +	+	+
	C7	+ +	+	+

	C8	+ + + +	+ + + +	+
**M-BA (D)**	D1	+ + +	-	-
	D2	+ +	-	-
	D3	-	-	-
	D4	+ +	-	-
	D5	+ +	+ +	+ +
	D6	+ +	+ +	+
	D7	+ +	+ + +	+
	D8	+ + +	+ + +	+
**H-BA (E)**	E1	+ + +	-	-
	E2	+ +	-	-
	E3	+ + +	-	-
	E4	+ + +	-	-
	E5	+ +	+	-
	E6	+ + +	+ +	+
	E7	+ +	+	+
	E8	+ + +	+ +	+
**rhG-CSF (F)**	F1	+ +	-	+ + +
	F2	+ +	-	+ +
	F3	+ +	-	+ +
	F4	-	-	+ + +
	F5	+ + +	-	+ + +
	F6	+	-	+
	F7	+	+ +	-
	F8	+	-	-

Notes: -: Slight lesion, lesion range less than 1/4 of the specified range. +: Mild lesion, lesion range more than 1/4 and less than 1/2 of the specified range. + +: Moderate

lesion, lesion range more than 1/2 and less than 3/4 of the specified range. + + +: severe lesion, lesion range more than 3/4 of the specified range.

Table 3: Effects of BA on Histopathologic and Morphological Changes of liver

Group	Animal number	Cell swelling	inflammatory cell infiltration	vacuolar degeneration	Extramedullary hematopoiesis	Venous wall thickening
**sham-IR(A)**	A1	-	-	-	-	-
	A2	++	-	-	++	-
	A3	+++	-	-	-	-
	A4	++	-	-	-	-
	A5	+	-	-	-	-
	A6	++	-	-	-	-
	A7	+++	-	-	-	-
	A8	+	-	-	-	-
**IR+vehicle(B)**	B1	+++	-	-	-	-
	B2	++	-	-	-	-
	B3	++++	-	-	-	-
	B4	-	-	-	-	-
	B5	++	-	+++	-	-
	B6	++++	-	+	-	-
	B7	++++	-	-	-	-
	B8	++++	-	+	-	-
**L-BA(C)**	C1	++	-	-	-	-
	C2	++	-	-	-	-
	C3	+++	-	-	-	-
	C4	+++	-	-	-	-
	C5	+++	-	-	-	-

	C6	++	-	+	-	-
	C7	++	-	+	-	-
	C8	+	-	-	-	-
**M-BA (D)**	D1	++	-	-	++	-
	D2	++++	-	-	-	-
	D3	-	-	-	++	-
	D4	+++	-	-	++	-
	D5	-	-	-	-	-
	D6	+++	-	-	-	-
	D7	+++	-	-	+	-
	D8	+	-	+	-	-
**H-BA (E)**	E1	-	-	-	-	-
	E2	++	-	++	+++	++
	E3	-	-	-	-	-
	E4	-	-	-	-	-
	E5	+++	-	-	++	-
	E6	++	-	-	-	-
	E7	++	-	-	-	-
	E8	++	-	-	-	-
**rhG-CSF (F)**	F1	+++	-	-	-	-
	F2	++	++	+++	-	-
	F3	+++	-	-	-	-
	F4	++	-	-	-	-
	F5	+++	-	-	-	-
	F6	++++	-	-	-	-
	F7	+	-	-	-	-

F8	-	-	-	-	-

Notes: -: Slight lesion, lesion range less than 1/4 of the specified range. +: Mild lesion, lesion range more than 1/4 and less than 1/2 of the specified range. + +: Moderate lesion, lesion range more than 1/2 and less than 3/4 of the specified range. + + +: severe lesion, lesion range more than 3/4 of the specified range.
